# Supreme Court of Illinois—Opinion

**Published:** 1884-07

**Authors:** 


### SUPREME COURT OF ILLINOIS.

OPINION FILED MAY 19, 1884.

*The People of the State of Illinois ex rel. Isaac A. Sheppard v. the Illinois State Board of Dental Examiners.*

PETITION FOR MANDAMUS.

POWER OF BOARD—LICENSE TO PRACTICE DENTISTRY.

Opinion by Scholfield, J.—It is provided by the first section of an act approved May 30, 1881, entitled "An act to insure the better education of practitioners of dental surgery, and to regulate the practice of dentistry in the State of Illinois," "that it shall be unlawful for any person who is not at the time of the passage of this act engaged in the practice of dentistry in this State, to commence such practice unless such person shall have received a diploma from the faculty of some reputable dental college, duly authorized by the laws of this State, or of some other of the United Sates, or by the laws of some foreign country, in which college or colleges there was at the time of the issue of such diploma, annually delivered a full course of lectures and instruction in dental surgery. * * *" And in the sixth section of the same act, after providing for examination, before the board of dental examiners, of all applicants for license to practice dentistry, is the following provision: "But said board shall, at all times, issue a license to any regular graduate of any reputable dental college without examination, upon the payment by such graduate, to the said board, a fee of one dollar. * * *"

Other provisions of the act prohibit any person to practice dentistry, without a license from the board, except such as are properly enrolled as having been practitioners at the time of the passage of the act. The contention of the relator is that the board of dental examiners have no power to decide what is or what is not a "reputable dental college;" that the law has, itself, defined what is a "reputable dental college" in providing that it shall be "duly authorized by the laws of this State, or some other of the United States, or by the laws of some foreign country, in which college * * there was at the time of the issue of such diploma, annually delivered a full course of lectures and instructions in dental surgery."

We are unable to appreciate the force of this position. The word "reputable" would seem to be used here to express the meaning ordinarily attached to it. If it had been intended that a diploma from any dental college, or a diploma from any dental college "duly authorized by the laws of this State, or some other of the United States, or by the laws of some foreign country, in which college * * there was at the time of the issue of such diploma, annually delivered a full course of lectures and instructions in dental surgery," we must presume the language would have so said. By using the word "reputable" we must presume the general assembly meant "reputable." And since it is not used as being the equivalent, and convertible, for the other requirements in regard to the college, but as in addition thereto, we must presume it was intended to be so construed. As a part of the current history of the times, and as an aid in arriving at the legislative intention, we know there were colleges of different kinds, authorized by the laws of States in which they were located, in which there were pretended to be annually delivered full courses of lectures and instructions upon the arts and sciences professed to be taught, that were not "reputable," because they graduated for money frequently without any reference to scholarship. A diploma from such an institution afforded no evidence of scholarship or attainments in its holder. It was a fraud, and deserved no respect from anybody. And it was as against such diplomas the law was intended to protect the public, and therefore required that the colleges be "reputable." Whether a college be reputable or not is not a legal question, but a question of fact. So also are the requirements in regard to the actual delivery of full courses of lectures and instructions. These questions of fact are, by the act, submitted to the decision of the board—not in so many words, but by the plainest and most necessary implication. Their action is to be predicated on the existence of the requisite facts, and no other tribunal is authorized to investigate them; and of necessity, therefore, they must do so. The act of ascertaining and determining what are the facts is in its nature judicial. It involves investigation, judgment, and discretion. The office of the writ of mandamus is, in general, to compel the performance of mere ministerial acts prescribed by law. It lies, however, also to subordinate judicial tribunals to compel them to act where it is their duty to act, but never to require them to decide in a particular manner. It is not like a writ of error or appeal, a remedy for erroneous decisions: Judges of Oneida Common Pleas *v.* People, 18, Wendell, 92. And as is said by the court in People ex rel. *v.* Com. Council of Troy, 78 N. Y., 33, this principle applies to every case where the duty, performance of which is sought to be compelled, is in its nature judicial, or involves the exercise of judicial power or discretion, irrespective of the general character of the officer or body to which the writ is addressed. A subordinate body can be directed to act, but not how to act, in a matter as to which it has the right to exercise its judgment. The character of the duty, and not that of the body or officers, determines how far performance of the duty may be enforced by mandamus.

Where a subordinate body is invested with power to determine a question of fact, the duty is judicial, and though it can be compelled by mandamus to determine the fact, it cannot be directed to decide it in a particular way, however clearly it be made to appear what the decision ought to be.

See also Kelly et al. *v.* City of Chicago, 62 Ill., 279.

Illustrations of the principle will be found in People *v.* Com. Council of Troy *supra;* Freemen *v.* Selectmen, etc., 34 Conn., 406; Hoole *v.* Kinkead, 17 Nevada, 217; Bailey *v.* Ewart, 52

Iowa, 111; Berryman *v.* Perkins, 55 Cal., 483; People *v.* Contracting Board, 27 N. Y., 378, and other cases cited in argument by the Attorney-General.

The demurrer does not admit here that the board of dental examiners found that the college at which the relator was graduated was reputable, although it does admit that to be the fact. But since the board cannot be compelled to decide the question that way, although the evidence might clearly sustain it in doing so, there is no ground for mandamus.

The demurrer must be sustained and the petition dismissed.

Petition dismissed."

The above decision is here given as the best way of replying to many inquiries as to the ground upon which it was rendered. It is a matter in which all, who have a just appreciation of dental education, will feel a deep interest; and it is desirable that it should receive the consideration its importance demands.—[Ed. Register.

